# Mouse bone marrow-derived mesenchymal stem cells inhibit leukemia/lymphoma cell proliferation *in vitro* and in a mouse model of allogeneic bone marrow transplant

**DOI:** 10.3892/ijmm.2015.2191

**Published:** 2015-04-21

**Authors:** NINGXIA SONG, LEI GAO, HUIYING QIU, CHONGMEI HUANG, HUI CHENG, HONG ZHOU, SHUQING LV, LI CHEN, JIANMIN WANG

**Affiliations:** 1 Department of Hematology, Institute of Hematology, PLA, Changhai Hospital, Second Military Medical University, Shanghai 200433, P.R. China; 2 Department of Hematology, Jinan Military General Hospital, Jinan 250031, P.R. China

**Keywords:** bone marrow-derived mesenchymal stem cells, leukemia, lymphoma, bone marrow transplantation, apoptosis, cell cycle

## Abstract

The allogeneic hematopoietic stem cell (HSC) transplantation of mesenchymal stem cells (MSCs) contributes to the reconstitution of hematopoiesis by ameliorating acute graft-versus-host disease (aGVHD). However, the role of MSCs in graft-versus-leukemia remains to be determined. In the present study, we co-cultured C57BL/6 mouse bone marrow (BM)-derived MSCs with A20 murine B lymphoma, FBL3 murine erythroleukemia and P388 murine acute lymphocytic leukemia cells. Cell proliferation, apoptosis, cell cycle progression and the amount of cytokine secretion were then measured using a Cell Counting kit-8, Annexin V/propidium iodide staining, flow cytometry and ELISA, respectively. We also established a model of allogeneic bone marrow transplantation (BMT) using BALB/c mice. Following the administration of A20 cells and MSCs, we recorded the symptoms and the survival of the mice for 4 weeks, assessed the T cell subsets present in peripheral blood, and, after the mice were sacrifice, we determined the infiltration of MSCs into the organs by histological staining. Our results revealed that the MSCs inhibited the proliferation of the mouse lymphoma and leukemia cells *in vitro*, leading to cell cycle arrest and reducing the secretion of interleukin (IL)-10. In our model of allogeneic BMT, the intravenous injection of MSCs into the mice injected wth A20 cells decreased the incidence of lymphoma, improved survival, increased the fraction of CD3^+^CD8^+^ T cells, decreased the fraction of CD3^+^CD4^+^ T cells and CD4^+^CD25^+^ T cells in peripheral blood, and ameliorated the manifestation of aGVHD. The results from the present study indicate that MSCs may be safe and effective when used in allogeneic BMT for the treatment of hemotological malignancies.

## Introduction

Interest in the therapeutic application of bone marrow (BM)-derived mesenchymal stem cells (MSCs) has increased in recent years, arising from the possibility that MSCs not only support hematopoiesis (1,2), but may also ameliorate graft-versus-host disease (GVHD) following allogeneic hematopoietic stem cell (HSC) transplantation (3). Allogeneic HSC transplantation is currently the only curative option for a number of hematological malignancies, although large numbers of MSCs are required to achieve clinical efficacy (4), and the effects of MSCs on tumor initiation, survival, progression and metastasis remain poorly understood. It is considered that MSCs suppress tumor growth. Otsu *et al* demonstrated that the direct inoculation of MSCs into subcutaneous melanomas induced apoptosis and abrogated tumor growth by inhibiting angiogenesis (5). Khakoo *et al* demonstrated that systemically injected MSCs reduced tumor growth in a model of Kaposi’s sarcoma through the inhibition of Akt (6), Zhu *et al* reported that human MSCs inhibited the proliferation of K562 cells by the secretion of Dickkopf-related protein 1 (DKK-1) (7). Wang *et al* recently reported that MSCs inhibit the proliferation of hepatic stellate cells through the inhibition of Toll-like receptor 4 (TLR4) signaling (8), and Menge *et al* reported that MSCs inhibit endothelial cell proliferation and angiogenesis through the modulation of the VE-cadherin/β-catenin signaling pathway (9). However, MSCs have also been reported to promote tumor growth. Galiè *et al* reported that MSCs co-implanted with cancer cells in syngeneic animals accelerated the appearance of tumors (10), possibly by promoting the angiogenic switch. MSCs have also been shown to increase the metastatic potential of breast cancer cell lines without altering primary tumor progression (11). Clearly, these data present a confusing picture of the contribution of MSCs to tumor formation, indicating that much study lies ahead in this field.

The aim of this study was to evaluate the therapeutic potential application of MSCs in allogeneic bone marrow transplantation (BMT) in hemotological malignanciess. First, we observed that in cell culture, C57BL/6 (B6) mouse MSCs inhibited the proliferation of leukemia and lymphoma cells, leading to cell cycle arrest and promoting apoptosis. In addition, in model of allogeneic BMT, transplanted MSCs inhibited the development of tumors induced by an injection of A20 B lymphoma cells. Our findings suggest that the clinical application of MSCs may contribute to the effectiveness of HSC transplantation in hematological malignancies.

## Materials and methods

### Mice

BALB/c (H-2^d^) and C57BL/6 (H-2^b^) (commonly known as B6 mice) mice (6–8 weeks old) were obtained from the Shanghai SLAC Laboratory Animal Co., Ltd. (Shanghai, China) and housed in plastic cages under specific pathogen-free conditions at the Institute for Animal Experiments, the Second Military Medical University (Shanghai, China). Chow and water were available at all times. The mice used in the experiments were gender- and age-matched. All animal experiments were performed following the approval of the Animal Care and Use Committee of the Changhai Hospital, Second Military Medical University (Shanghai, China).

### Preparation of MSCs

The B6 mice were sacrificed by cervical dislocation, and the femurs and tibias were removed and cleaned of all connective tissue. BM cells were collected by flushing the femurs and tibias with medium using a 26-gauge needle (Shandong Weigao Group Medical Polymer Co., Ltd., Shandong, China), filtered, and washed twice by centrifugation at 1,500 rpm for 6 min. The cells were cultivated in 21-cm^2^ plates (BD Biosciences, Franklin lakes, NJ, USA) at 10^6^ cells/cm^2^ in Dulbecco’s modified Eagle’s medium (DMEM) (Gibco, Grand Island, NY, USA), supplemented with 10% FCS (Gibco), 100 IU/ml penicillin, 100 *µ*g/ml streptomycin (Sigma, St. Louis, MO, USA) and 2 mM L-glutamine (Sigma) at 37°C in 5% CO_2_. After 48 h, the non-adherent cells were removed and the medium was replenished every 3 days. At 70–80% confluence, the adherent cells were incubated for 2–3 min at 37°C with a 0.05% trypsin solution containing 0.02% EDTA (PAA). Trypsin was neutralized by the addition of fresh complete medium, and the cells were then harvested, and re-plated at a ratio of 1:2. MSCs were identified by detecting the expression of CD29, CD44, CD90.2, stem cell antigen-1 (Sca-1), fms-related tyrosine kinase 1 (Flk-1), c-kit and major histocompatibility complex (MHC)-I, and by the absence of the expression of CD34, CD45, MHC-II, determined by flow cytometry (using a flow cytometer, BD Biosciences). All antibodies used [PE-CD29 (12-0291), PE-CD44 (12-0441), APC-CD90.2 (17-0902), PE-Sca-1 (15-5981), PE-Flk-1 (12-5821), FITC-c-Kit (11-1171), PE-MHC-I (12-5999), Biotin-CD34 (13-0341), FITC-CD45 (11-0451), PE-MHC-II (12-5322)] were purchased from eBioscience, Inc. (San Diego, CA, USA). MSCs at passages 3–10 were used in the experiments.

### Cell culture

The A20 B lymphoma cell line (H-2^d^), FBL3 erythroleukemia cell line (H-2^b^), P388 acute lymphocytic leukemia cell line (H-2^k^) (all from the Cell Resource Center of the Shanghai Institute of Life Science of the Chinese Academy of Sciences) were kept in liquid nitrogen. The cells were maintained in RPMI-1640 medium (Gibco) containing 10% heat-inactivated newborn calf serum (NCS) (purchased from Gibco), 100 U/ml penicillin and 100 *µ*g/ml streptomycin in a humidified atmosphere of 5% CO_2_ at 37°C.

### Co-culture of MSCs and leukemia and lymphoma cell lines

The mouse MSCs were harvested and pre-treated with 25 *µ*g/ml mitomycin C (MMC) (Sigma) at 37°C for 20 min, then washed twice with PBS and resuspended in DMEM supplemented with 10% FCS, 2 Mm glutamine, 100 U/ml penicillin and 100 *µ*g/ml streptomycin, and plated into a 96-well plate at 2×10^3^, 5×10^3^, 2×10^4^ and 5×10^4^ cells/well in 0.2 ml medium. After 10 h, the MSCs had adhered to the plates, and the medium was removed. The A20, FBL3, P388 cancer cell lines were added to the wells to achieve an MSC:cancer cell ratio of 1:10, 1:4, 1:1, or 1:0.4 in 0.2 ml medium, and co-cultured for 48 h. The cancer cell lines were cultured alone or with the MSCs for 24, 48 and 72 h. The non-adherent cells (leukemia and lymphoma cells) were collected to assess cell proliferation and the cell cycle.

To determine whether the MSCs inhibit A20 cell proliferation through the Akt protein kinase pathway, an Akt inhibitor (Cat. no. 124005; Calbiochem, La Jolla, CA, USA) was added to the A20 cells co-cultured with the MSCs at a ratio of 1:1 at a final concentration of 5 *µ*M. After 48 h, the cells were collected for further analysis.

### Assessment of cell proliferation

The floating A20 cells cocultured with the MSCs were isolated from the MSCs which were attached to the Transwell membrane (Corning, Inc., Corning, NY, USA). Cell viability was assessed using a Cell Counting kit-8 (CCK-8) (Dojindo Laboratories, Kumamoto, Japan). A total of 6 wells was included in each sample and MSCs were used as controls. CCK-8 was added 4 h before the end of the culture time. Wells without cells were set as blanks. The absorbance at 450 nm was measured using a Universal Microplate Spectrophotometer (Thermo Fisher Scientific, Inc., Waltham, MA, USA). The relative proliferation rate (%) of the cancer cells was calculated as follows: (OD_450_ of cancer cell lines with MSCs - OD_450_ of MSC control)/OD_450_ of cancer cell lines ×100.

### Assessment of cell cycle progression and early apoptosis

To determine the effect of MSCs on cancer cell apoptosis and cell cycle distribution, the MSCs were seeded into 6-well plates at 1×10^5^ cells/well. After 10 h, the MSCs had adhered to the plates, the medium was removed followed by the addition of 1×10^5^/well of cancer cells After 72 h, the floating cancer cells were harvested. The apoptosis of the cancer cells was analyzed by Annexin V/propidium iodide (PI) staining (eBio-science, Inc.) according to the manufacturer’s instructions. For cell cycle analysis, 1 million cancer cells were fixed with 70% cold ethanol at 4°C overnight, washed with PBS twice, and 10 *µ*g (1 mg/ml) RNase and 0.3 ml (50 *µ*g/ml) PI (Sigma) were then added to the cell suspension for 30 min. Cell fluorescence was assessed using a BD FACSCalibur Flow Cytometer and analyzed using CellQuest software (BD Biosciences). Data were analyzed using ModFit software.

In order to determine the expression of cell cycle- and apoptosis-related genes, the mRNA expression of *p21* and *caspase-3* was analyzed by reverse transcription-quantitative PCR (RT-qPCR). Total RNA was extracted using TRIzol reagent (Invitrogen Life Technologies, Carlsbad, CA, USA), and the concentration and purity of the RNA were estimated by optical density measurements. For PCR, the cDNA samples were standardized based on the mRNA expression of β-actin. Total RNA (500 ng) was reverse transcribed and amplified using the Takara PrimeScript One Step RT-PCR kit [Takara Biotechnology (Dalian) Co., Ltd., Liaoning, China]. RT-PCR was performed using the following primers for 43 cycles at 95°C for 2 min, at 95°C for 13 sec, and at 58°C for 1 min: *p21* forward, 5′-CCCGAGAACGGTGGAACT-3′ and reverse, 5′-AGAGGGCAGGCAGCGTAT-3′; *caspase-3* forward, 5′-ATGTCATCTCGCTCTGGT-3′ and reverse, 5′-TCTG TTTCTTTGCGTGGA-3′; and *β-actin* forward, 5′-GCCA TGTACGTAGCCATCCA-3′ and reverse, 5′-AACCGCT CATTGCCGATAGT-3′. Quantitative (real-time) PCR (qPCR) was performed using an ABI PRISM Sequence Detection System 7500 (Applied Biosystems, Foster City, CA, USA) with the QuantiTect™ SYBR-Green PCR kit (Qiagen, Hilden, Germany). Triplicate wells were averaged and the relative quantities of *p21* and *caspase-3* were then calculated using the comparative Ct method. The mRNA expression levels were normalized using the β-actin control 1/2^ΔCT^ value. The relative gene expression was calculated in triplicate as follows: the amp lication of (cancer cells co-cultured with MSCs)/(cancer cells alone).

### Measurement of extracellular cytokine and intracellular interleukin (IL)-10 levels by ELISA

The A20 cells were cultured alone or with MSCs at a ratio of 1:1. After 24, 48 or 72 h the supernatants were harvested, and the concentrations of IL-10, transforming growth factor (TGF)-β, tumor necrosis factor (TNF)-α, and interferon (IFN)-γ were measured by ELISA (eBioscience, Inc.) according to the manufacturer’s instructions. Intracellular IL-10 staining was performed on the cells after 48 h of co-culture using the anti-mouse IL-10 staining kit according to the directions for use (eBioscience, Inc.). The cells were collected, washed with PBS and resuspended in PBS. Mouse FITC-CD19 antibody (11-0193) was added, followed by the addition of fixation and penetrating solutions. Thereafter, mouse PE-IL-10 antibody (12-7101) was added. After 20 min, the cells were washed with penetrating solution and resuspended in PBSC. The numers of CD19^+^IL-10^+^ cells were assessed using a BD FACSCalibur Flow Cytometer and analyzed using CellQuest software (BD Biosciences).

### Allogeneic BMT in mice injected with A20 B lymphoma cells

The BABL/c recipients were conditioned with a lethal 7-Gy (^60^Co, 80c Gy/min) dose of total body irradiation, 6 h before transplantation. The mice were divided into 5 groups according to the intravenous infusion of cell types as follows: i) the control group (n=10), no cells but only PBS was administered; ii) the BM group (n=10), 1×10^7^ donor BM cells were injected; iii) the BM-MSC group (n=10), 1×10^7^ donor BM cells and 5×10^5^ MSCs were injected; iv) the A20 group (n=17), 1×10^7^ donor BM cells and 1×10^4^ A20 cells were injected; and v) the MSC-A20 group (n=17), 1×10^7^ donor BM cells, 1×10^4^ A20 cells and 5×10^5^ MSCs were injected. The survival and appearance of the mice were monitored daily, and body weight was measured every other day. Kaplan-Meier survival curves were established for each group. Mice suffering from advanced-stage disease were sacrificed for histological examination, and this event was considered as a death in the survival curve.

In order to assess the homing of the allogenic MSCs, we injected the above-mentioned mice with MSCs stained with a fluorescence marker. The MSCs were resuspended in DMEM supplemented with 10% FBS and washed twice with PBS. Fluorescence labeling was performed by incubating 10^7^ MSCs in 1 ml Diluent C supplemented with freshly prepared 16 *µ*M PKH67 membrane linker (Sigma) for 5 min at room temperature, and the staining reaction was then terminated by the addition of an equal volume of serum. Donor BM cells (1×10^7^) were injected intravenously with or without 5×10^5^ murine MSCs. The mice were sacrificed 24 h or 7 days following transplantation for the frozen section examination of the tissues of the heart, lungs, liver, spleen, small intestine and kidneys. The 8 *µ*m cryosections were then observed under a fluorescence microscope (Leica, Wetzlar, Germany).

### Analysis of T cell subsets

On days 7 and 14 following transplantation, 100 *µ*l peripheral blood was collected from the retro-orbital vein of each mouse under ether anesthesia in a heparinized tube, and lymphocyte subset analysis was performed using fluorochrome-conjugated anti-mouse monoclonal antibodies (mAbs): murine PE-cy5.5-CD3e, FITC-CD4, PE-CD8, APC-CD25 and PE-MHC-I (H-2K^b^) (eBioscience, Inc.), followed by 30 min of incubation and then by erythrocyte lysis using BD FACS Lysing Solution (BD Biosciences) according to manufacturer’s instructions. The samples were washed twice in PBS and the pellet was resuspended in 200 *µ*l of PBS. Approximately 20,000 events/sample were acquired on a BD FACSCalibur Flow Cytometer and analyzed using CellQuest software (BD Biosciences).

### Histological examination

Four weeks after allogeneic BMT, the remaining mice were sacrificed. The livers, small intestine, lungs and spleen were obtained in order to evaluate histological changes. The tissue samples were fixed in 4% formaldehyde solution for several days and embedded in paraffin, and 5-*µ*m sections were stained with hematoxylin and eosin (H&E) for histological examination.

### Statistical analysis

Unless otherwise stated, the experiments were repeated at least 3 times. Statistical analysis was performed using Excel (Microsoft) and SPSS 11.0 statistical analysis software (SPSS, Inc., Chicago, IL, USA). All experimental quantitative data are expressed as the means ± standard deviation. A Student’s t-test or covariance analysis were performed for statistical analysis. Percentages were compared using the Chi-square test (Fisher’s exact test). Kaplan-Meier survival curves were established for each group. A value of P<0.05 was considered to indicate a statistically significant difference.

## Results

### MSCs inhibit the proliferation of leukemia and lymphoma cells

**Figure 1:**
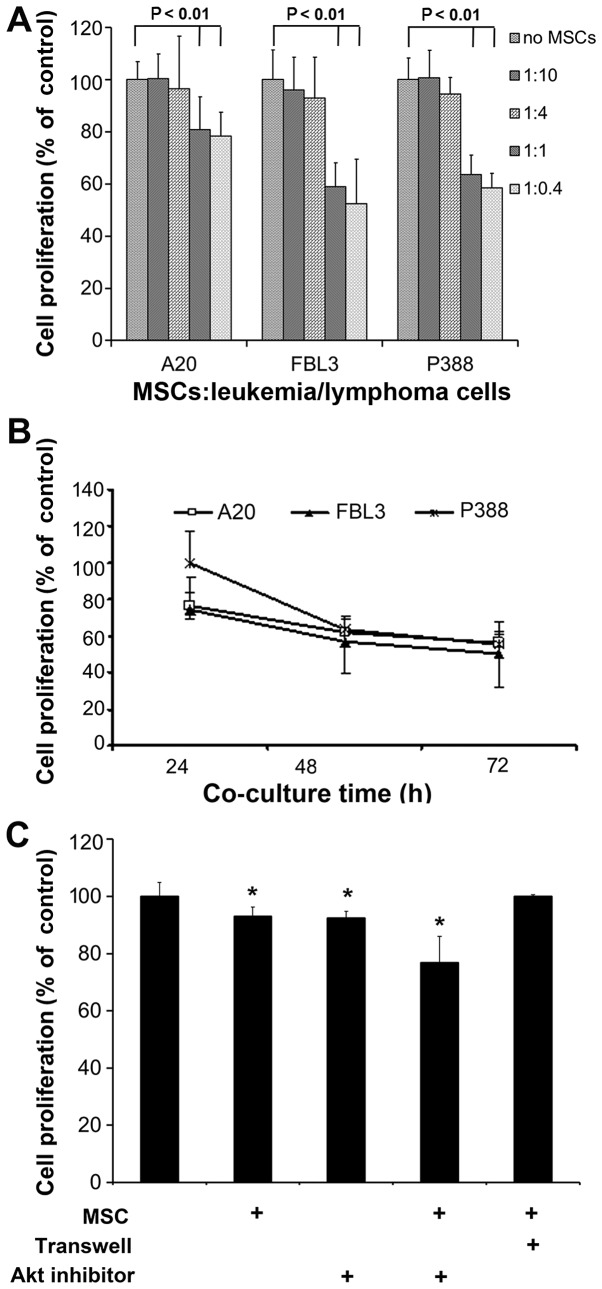
Mesenchymal stem cells (MSCs) inhibit the proliferation of malignant cells of various hematopoietic origins. (A) The cells (2×10^4^; A20 B lymphoma, FBL3 erythroleukemia and P388 acute lymphocytic leukemia cells) were cultured in the presence or absence of the indicated numbers of MSCs in 96-well plates for 48 h. Cell proliferation was assessed using a Counting kit-8 (CCK-8) assay during the final 4 h of culture. (B) The leukemia and lymphoma cells (2×10^4^) were cultured in 96-well plates in the presence of 2×10^4^ MSCs for the indicated periods of time. (C) A20 cells (2×10^4^) were cultured in 96-well plates. MSCs (2×10^4^) were added directly to the A20 cells or on the other side of a Transwell insert, and the plates were co-cultured for 48 h in the presence or absence of an Akt inhibitor (5 *µ*M). A20 cell proliferation was measured using a CCK-8 assay during the final 4 h of culture. The results are shown as a percentage of cell proliferation in comparison with control lymphoma cell proliferation. Results are expressed as the means ± SD of 3 independent experiments. ^*^P<0.05 indicates statistical significance when compared with the control group.

We investigated the effects of the MSCs on the prolif-erative activity of leukemia and lymphoma cells of different lineages. The A20 B lymphoma cells (H-2^d^), the FBL3 erythroleukemia cells (H-2^b^) and the P388 acute lymphocytic leukemia cells (H-2^k^) were cultivated with the MSCs for 48 h. When the B6 MSCs were added to the culture in concentrations equivalent to 1 MSC to 1 or 0.4 leukemia cells, the proliferation of the leukemia and lymphoma cells was inhibited; however when the leukemia and lymphoma cells were in excess, the proliferation was not inhibited (Fig. 1A). Furthermore, the anti-proliferative effects of the MSCs increased with increasing co-culture times (Fig. 1B), but these effects were lost when the MSCs and leukemia/lymphoma cells were separated by a permeable membrane (Fig. 1C), indicating that the MSCs inhibited the proliferation of the leukemia and lymphoma cells in a contact-dependent manner. In addition, although it has previously been reported that human MSCs suppress tumor development by inhibiting target-cell Akt activity (6), we did not find that Akt inactivation affected the proliferation of the A20 cells co-cultured with MSCs (Fig. 1C), suggesting that the inhibition of lymphoma cell proliferation by mouse MSCs may not involve the inhibition of Akt.

### MSCs induce early apoptosis and cell cycle arrest in leukemia and lymphoma cells in a contact-dependent manner

**Figure 2:**
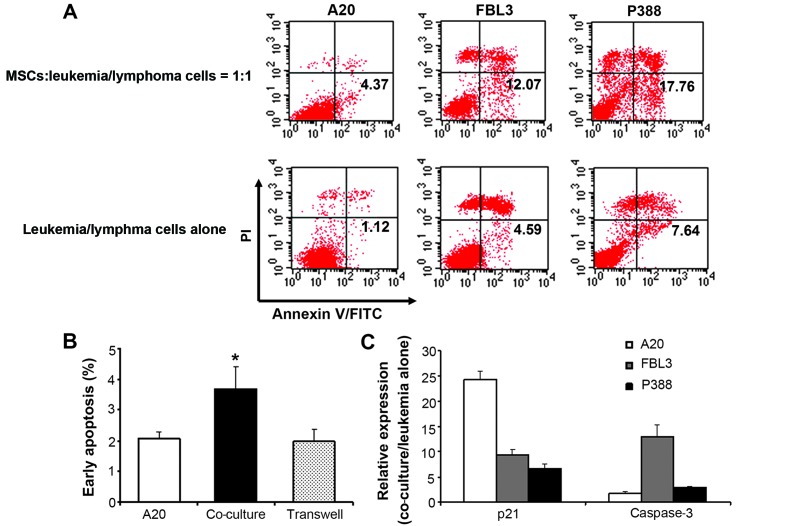
Mesenchymal stem cells (MSCs) induce the early apoptosis of leukemia and lymphoma cells. (A) Cells (1×10^5^; A20 B lymphoma, FBL3 erythroleukemia and P388 acute lymphocytic leukemia cells) were cultured for 72 h alone or in the presence of MSCs at ratio of 1 MSC to 2 leukemia/lymphoma cells. The cells were then harvested and analyzed by Annexin V and propidium iodide (PI) double staining. (B) A20 cells (1×10^5^) were cultured under 3 different conditions in 6-well plates for 72 h: with or without MSCs (ratio 1:1, respectively); some were physically separated from teh MSCs using a Transwell system. (C) *p21* and *caspase-3* mRNA expression in A20, FBL3, and P388 cells detected by RT-qPCR. *p21* and *caspase-3* mRNA expression was increased in the cells in co-culture relative to culture alone. The data are expressed as the means ± SD of 3 separate experiments. ^*^P<0.05 indicates statistical significance.

**Figure 3:**
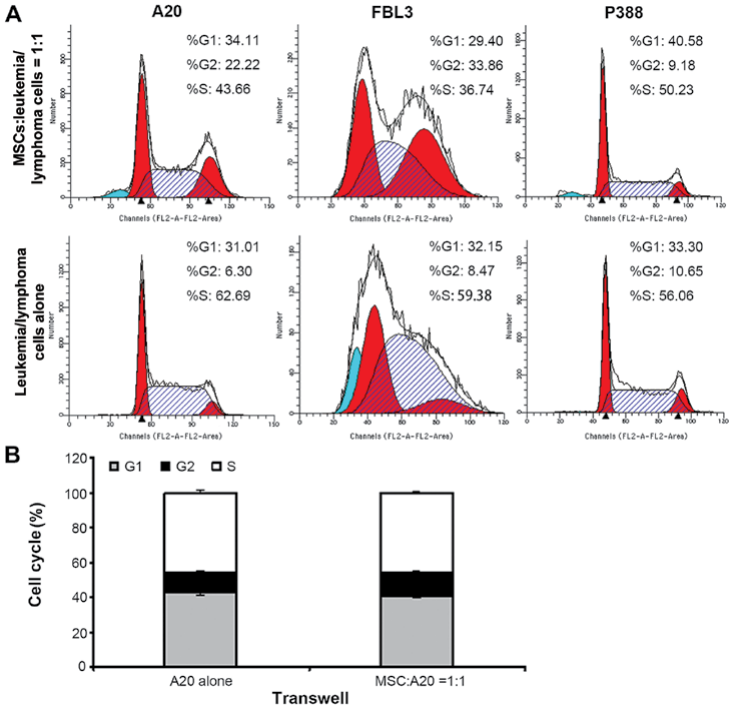
Mesenchymal stem cells (MSCs) induce cell cycle arrest in leukemia and lymphoma cells. (A) Cell cycle distribution was analyzed after the cells (A20 B lymphoma, FBL3 erythroleukemia and P388 acute lymphocytic leukemia cells) were co-cultured with the MSCs for 72 h at a ratio 1:1. (B) A20 cells (2×10^4^) were cultured alone or with 2×10^4^ MSCs separated using a Transwell system.

When the A20 B lymphoma cells, the FBL3 erythroleukemia cells and the P388 acute lymphocytic leukemia cells were co-cultured with the MSCs for 72 h, the proportion of apoptotic cells, as measured by Annexin V and PI staining, was significantly increased (Fig. 2A and B). The proportion of cells in the G_0_/G_1_ phase was also significantly higher in the cells incubated with the MSCs than in those incubated alone, while the proportion of cells in the S phase was significantly decreased following co-culture with the MSCs for 72 h (Fig. 3A and Table I). When the MSCs were physically separated from the A20 cells using a Transwell system, they no longer influenced the early apoptotic rate of the A20 cells (Fig. 2B) or the cell cycle (Fig. 3B), suggesting that MSCs influence the cell cycle and apoptosis of lymphoma cells in a contact-dependent manner.

Furthermore, we assessed the mRNA levels of the cell cycle negative regulator, *p21*, and the apoptosis-associated protease, *caspase-3*. qPCR revealed that incubation with the MSCs induced a significant upregulation in the mRNA levels of *p21* and *caspase-3* in the leukemia and lymphoma cells when compared with the cancer cells cultured alone (Fig. 2C).

### MSCs inhibit the secretion of cytokines from A20 cells

**Figure 4:**
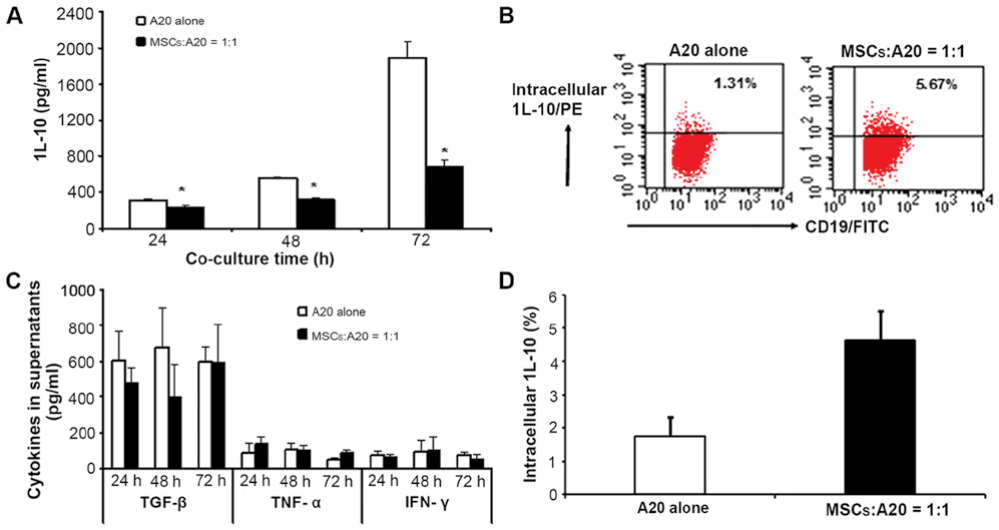
Co-culture of A20 B lymphoma cells and mesenchymal stem cells (MSCs) causes a decrease in the levels of interleukin (IL)-10 in the supernatant. A20 cells (1×10^5^) were cultured in 6-well plates in the presence or absence of MSCs at ratio 1:1 for 24, 48, or 72 h. (A) IL-10 levels in A20 culture supernatants were measured by ELISA in the presence or absence of MSCs for 72 h. (B) The fraction of CD19-positive A20 cells expressing intracellular IL-10 was measured by flow cytometry. (C) The levels of transforming growth factor (TGF)-β, tumor necrosis factor (TNF)-α and interferon (IFN)-γ in the culture supernatants were measured by ELISA. (D) The fraction of cells containing intracellular IL-10 was measured by flow cytometry. Results are expressed as the means ± SD of 3 independent experiments. ^*^P<0.05 indicates statistical significance.

As cytokines play an important role in the regulation of adaptive and innate immune responses to tumors (12–14), we measured the contents of cytokines in the A20 cells incubated in the presence or absence of MSCs. The A20 cells were found to express low levels of TNF-α and IFN-γ, moderate levels of TGF-β, and high levels of IL-10 (Fig. 4A and B). When co-cultured with the MSCs at a ratio of 1:1, the levels of IL-10 in the supernatant were significantly decreased, and the inhibitory effects of the MSCs on IL-10 secretion became more prominent with time (Fig. 4A). However, the levels of TGF-β, TNF-α, and IFN-γ in the supernatant were not affected by the presence of MSCs (Fig. 4C).

To determine whether the decrease in the content of IL-10 in the co-culture supernatant was related to IL-10 secretion, we measured the intracellular IL-10 levels in the A20 cells. When the A20 cells were co-cultured with the MSCs for 48 h, the fraction of cells in which intracellular IL-10 could be detected increased from 1.74±0.59% to 4.64±0.89%, suggesting that MSCs influence the capacity of lymphoma cells to release IL-10 (Fig. 4B and D).

### MSCs inhibit A20 lymphoma cell growth in the mouse model of allogeneic BMT

**Figure 5:**
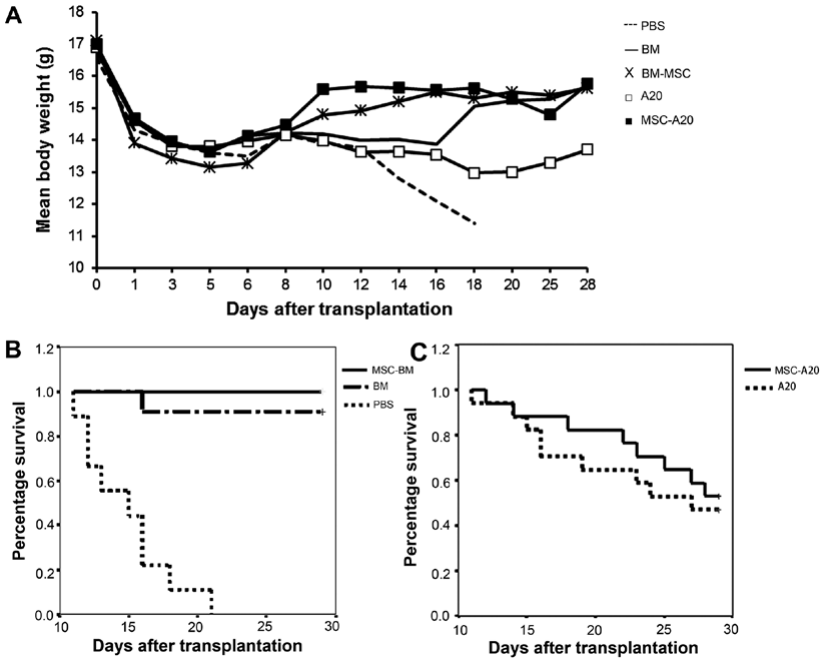
Mesenchymal stem cells (MSCs) increase the weight and extend the survival of mice in a model of allogeneic bone marrow transplantation (BMT). (A) Mice were weighed following BMT, and the mean wild-type curves ± SEM were established for the mice receiving PBS (–, n=10), bone marrow (BM) cells alone (−, n=10), BM cells supplemented with MSCs (×, n=10), BM cells plus A20 cells (◻, n=17), or BM cells with A20 cells plus MSCs (■, n=17). (B and C) Results are represented as a Kaplan-Meier survival curve. (B) There was significant difference between the PBS group and the other 2 groups (BM and BM-MSC group) (P<0.01). (C) Lethally irradiated BABL/c mice were transplanted with 1×10^7^ BM cells and 1×10^4^ A20 cells with or without 5×10^5^ MSCs. The survival rate in the 2 groups was not significant, but the the time of death of the mice injected with MSCs and A20 cells was delayed.

**Figure 6:**
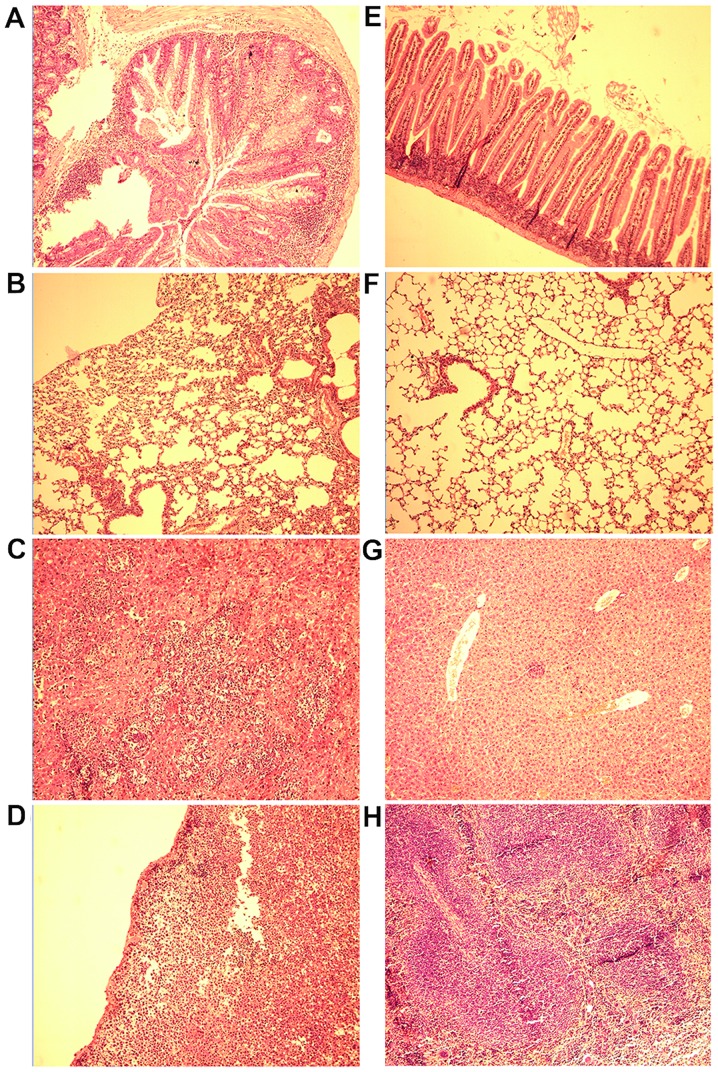
Histological analysis of the mice injected with A20 cells alone and mesenchymal stem cell (MSC) plus A20 cells on day 28 following transplantation. Mice in the (A-D) A20 group and (E-H) MSC-A20 group were sacrificed, and the small intestine, lungs, liver, and spleen were removed, fixed, paraffin-embedded, and sectioned. Paraffin-embedded 5-*µ*m tissue sections were stained with hematoxylin and eosin. (A) Small intestinal mucosa presented necrosis, defluxion and vacuolar degeneration. (B) Collapse of pulmonary alveoli. (C) Large tumor nodule infiltrating the liver, crushing normal liver tissues. (D) A20 cells infiltrated the spleen. (E-F) Normal small intestine and lung structures. (G-H) Normal liver and spleen structures. Objective lens: magnification, ×10.

MSCs have been reported to both promote (15) and abrogate (5) tumor growth *in vivo*. In this study, in order to investigate the effect of MSCs in a model of allogeneic BMT, we implanted A20 B lymphoma cells into mice. Lethally irradiated BABL/c female mice were either injected with PBS (n=10), grafted with 1×10^7^ B6 BM cells (n=10), 1×10^7^ donor BM cells and 5×10^5^ MSCs (n=10); 1×10^7^ donor BM cells and 1×10^4^ A20 cells (n=17); or 1×10^7^ donor BM cells, 1×10^4^ A20 cells and 5×10^5^ MSCs (n=17). Ninety percent of the grafted mice in the BM group and 100% in the BM-MSC group survived for >28 days, while the ungrafted animals died before day 21 (Fig. 5B). When 1×10^4^ A20 cells were injected, the mice in the BM group exhibited a characteristic infiltration of leukemia/lymphoma cells presenting as paralysis and splenohepatomegalia. The mice administered the A20 and MSCs did not exhibit paralysis, but exhibited mild splenohepatomegalia. The mice injected with A20 cells also exhibited a hunched posture, dull fur and slight diarrhea, and the histological examination revealed necrosis, defluxion, vacuolar degeneration of the small intestinal mucosa, and atrophy and collapse in the alveolae. These symptoms were less severe in the mice that also received MSCs (Fig. 6), and the mean body weight was significantly higher in the mice that received MSCs in addition to A20 cells from 8 days after irradiation (Fig. 5A). Excluding those mice that died of complications associated with irradiation, the mice administered the A20 cells and MSCs survived longer than the mice administered only A20 cells (Fig. 5C).

To investigate the effect of MSCs on the proliferation of A20 cells *in vivo*, we compared the incidence of lymphoma between the mice injected with MCCs and those that were not, and found that the injection of MSCs significantly decreased the incidence of lymphoma in the mice injected with A20 cells (P<0.05, Table II).

### Tracing the PKH26-labeled MSCs in the allogeneic BMT setting

Since our results revealed that the B6 MSCs inhibited the proliferation of the A20 cells, increased early apoptosis and led to cell cycle arrest *in vitro*, we wished to determine whether intravenously injected mouse MSCs can home to A20 lymphoma-infiltrated organs (such as the spleen and liver). On day 0, 1×10^6^ MSCs were injected with 1×10^7^ BM cells and 1×10^4^ A20 cells into the tail vein of the irradiated BABL/c mice. To track the distribution of the MSCs within the mouse over time, we labeled the MSCs with PKH26 (a fluorescent membrane linker) to allow for the easy identification in histopathological sections. We have previously established that PKH26-labeling efficiency is almost 100% and does not affect the growth of MSCs *in vitro* (data not shown), and a similar approach has been previously demonstrated to effectively track the *in vivo* location of MSCs without alterating cellular functions (16).

**Figure 7:**
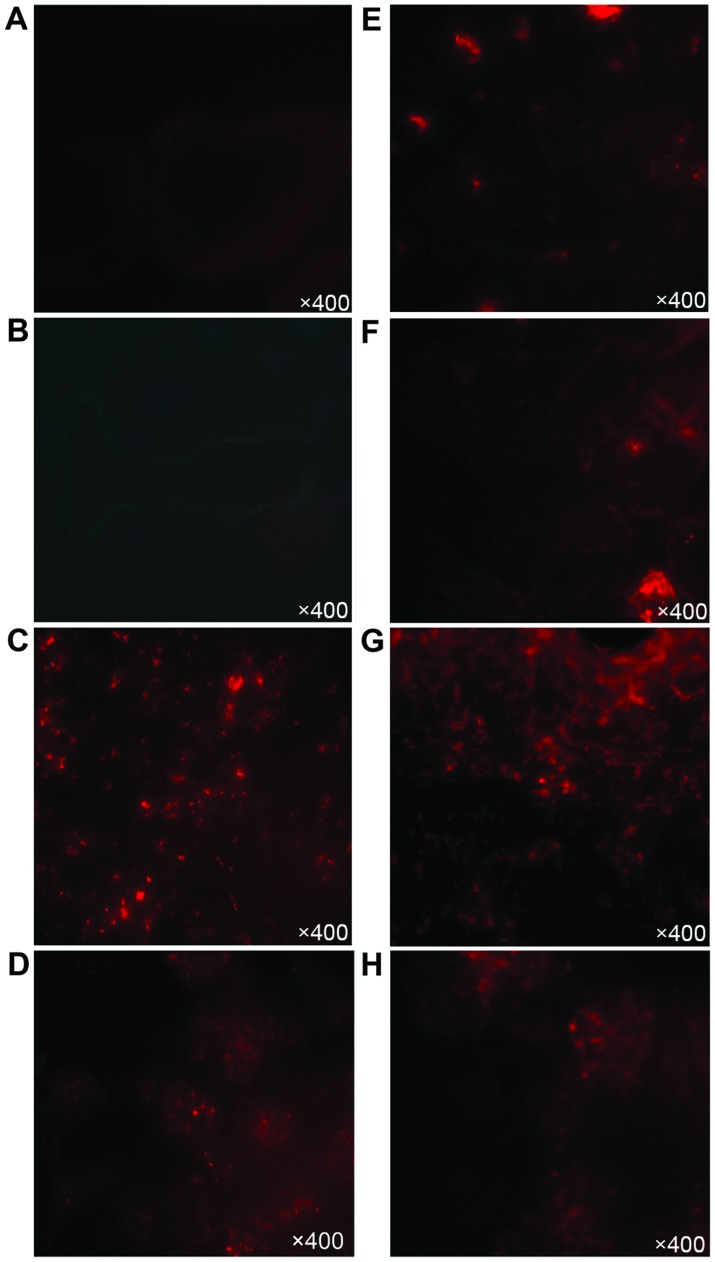
Cyosections of tissues at (A-D) 24 h or (E-H) 7 days after transplantation. We observed no mesenchymal stem cella (MSCa) in the (A) small intestine and (B) lunga, but numerous PKH26-labeled MSCs in the (C) spleen and (D) kidneya at 24 h post-grafting; (E-H) Fluorescence microscopic images confirmed the presence of PKH26-labeled MSCa in the (E) liver, (F) lunga, (G) spleen and (H) kidneya 7 days after transplantation, and a large number of PKH26-labeled MSCs in the spleen.

Twenty-four-hours post-grafting, the labeled MSCs were observed to be diffusely distributed in the spleen and kidneys (Fig. 7C and D), while no MSCs were observed in the heart, liver, small intestine (Fig. 7A) or lungs (Fig. 7B). On day 7, we could still observe numerous MSCs within the spleen and kidneys (Fig. 7G and H), which appeared to maintain the elongated, fibroblast-like appearance that they adopt in culture. Furthermore, on day 7, the donor MSCs were present in the liver and lungs of the grafted mice. This indicated that following their infusion, the MSCs mainly migrated to the tumor sites (spleen and liver) and the damaged tissues (lungs and kidneys). We were, however, surprised not to find any MSCs in the easily injured small intestine 24 h or 7 days post-grafting.

### Effect of MSCs on peripheral blood T cell subsets in the model of allogeneic BMT

When blood was taken from the mice on day 7 post-grafting, we found that the percentage of CD3^+^CD8^+^ T cells was 32.5±7.29% higher in the peripheral blood of the mice administered MSCs and A20 cells in comparison to those administered only A20 cells, while the percentages of CD3^+^CD4^+^ T cells and CD4^+^CD25^+^ T cells were significantly decreased (Table III). However no significant differences were observed in the T cell subsets on day 14 following transplantation (Table III).

## Discussion

MSCs have been reported to suppress immune responses in some contexts, and to enhance them in others [reviewed in (17)]. While MSCs show great promise in the treatment of autoimmune diseases, such as GVHD (3), Crohn’s disease (18) and multiple sclerosis (19), MSCs can also contribute negatively in diseases, such as cancer, and the effect of MSCs on the proliferation of hematopoietic cells has not been well documented (20,21). In this study, we found that MSCs from B6 mice inhibited the proliferation of leukemia and lymphoma cells in a dose- and time-dependent manner *in vitro*. This finding is consistent with that of a previous study demonstrating that MSCs exhibit a similar anti-proliferative activity in cancer cells of hematopoietic and non-hematopoietic origin (22).

The mechanisms through which MSCs inhibit the proliferation of leukemia/lymphoma cells have not been well characterized. Otsu *et al* found that in a Matrigel angiogenesis assay, high numbers of MSCs increased the production of reactive oxygen species, inhibiting capillary growth, and abrogating tumor growth (5). Similar to the findings of the study by Lu *et al* (20), we demonstrated that MSCs upregulated *p21* and *caspase-3* mRNA expression in leukemia and lymphoma cells, increasing the fraction of cells undergoing early apoptosis, and leading to cell cycle arrest at the G_0_/G_1_ phase, thus decreasing the fraction of leukemia and lymphoma cells in the S phase. In contrast to the results reported in the study by Khakoo *et al*, who found that human MSCs inhibited the proliferation of Kaposi’s sarcoma cells by inhibiting the *in vitro* activation of the Akt protein kinase (6), we did not find that the inhibitory effects of MSCs were affected by the addition of an Akt inhibitor, indicating that the effects of MSCs on lymphoma cells are not medaited through the Akt pathway.

BALB/c-derived B lymphoma A20 cells express high levels of IL-10 and this cytokine may contribute to their immune evasion by affecting various arms of the immune system, including Treg cells and dendritic cells (DCs) (23). In this study, we found that A20 cells secreted high levels of IL-10 and moderate levels of TGF-β, but very low levels of TNF-α and IFN-γ. The levels of IL-10 in the supernatant of A20 cell cultures were significantly decreased by co-culture with MSCs in a time-dependent manner. Furthermore, when co-cultured with the MSCs, the fraction of A20 cells expressing intracellular IL-10 significantly increased, suggesting that MSCs inhibited the secretion of IL-10 by A20 cells.

We found that both the survival rates and body weights of the mice increased significantly following transplantation of the MSCs, and the symptoms were significantly ameliorated in the mice were injected with the MSCs. Previous studies on the use of MSCs in murine models of tumors have focused on the NOD-SCID or athymic nude mouse model (20,24) or subcutaneous model (15,25). Ramasamy *et al* reported that MSCs formed a cancer stem cell niche in which the potential of cancer cells to proliferate is preserved, sustaining the malignant process (22). However, in their study, the experiments were performed using immunodeficient nude mice, and do not reflect the situation of autologous tumor development. Djouad *et al* reported that the subcutaneous injection of B16 melanoma cells led to tumor growth in allogeneic recipients only when MSCs were co-injected, which were related with the immunosuppressive properties in mixed lymphocyte reaction (15). However, these mouse tumor models do not represent the leukemia/lymphoma environment well. By contrast, in this study, we observed the amelioration of aGVHD in mice in a model of BMT, and found the incidence of lymphoma in the liver/spleen to be lower in the mice administered MSCs. Thus, consistent with the results of the study by Baron *et al*, who reported in a clinical study that the co-transplantation of MSCs prevented death from GVHD without abrogating graft-versus-tumor effects after HLA-mismatched allogeneic transplantation following non-myeloablative conditioning (26), our results support the hypothesis that allogeneic BM-derived MSCs control aGVHD without increasing the incidence of lymphoma. To the best of our knowledge, this study represents the first report aiming to evaluate the effects of MSCs on the development of lymphoma/leukemia cells in an allogeneic BMT model of minimal residual leukemia. Although the A20 lymphoma model used in this study does not fully capitulate the physiological circumstances of leukemia/lymphoma cell proliferation in humans, it provides some insight as to whether the systemic injection of allogeneic BM-derived MSCs in a BMT setting may influence the growth of malignant cells.

We also observed the abrogation of T cell subsets in the peripheral blood of mice. The percentage of CD3^+^CD8^+^ T cells increased and the fraction of CD3^+^CD4^+^ T cells and CD4^+^CD5^+^ T cells decreased in the mice administered MSCs 7 days following transplantation. However, these differences had disappeared by day 14. This perhaps reflects the reduction in the numbers of A20 cells *in vivo* at an early stage. We also detected large numbers of MSCs, not only in the target organs of GVHD, but also in the spleen, and furthermore detected some MSCs in the livers of the recipients 7 days after grafting. We also found that the inhibition of A20 cell proliferation by MSCs *in vitro* was contact-dependent, suggesting that cell-cell contact may be necessary for MSCs to inhibit the leukemia/lymphoma cell cycle and cell proliferation. It has also been recently reported that the capacity of MSCs to inhibit hepatic stellate or endothelial cell proliferation is also contact-dependent (8,9). The present study in combination with previously reported results suggests that the involvement of a communication microenvironment between MSCs and leukemia cells is required. We also thus speculate that it is the MSCs present in the spleen and liver that directly inhibit the proliferation of A20 cells, which are also primarily present in the spleen and liver of the recipient mice.

In conclusion, we found that the inhibition of the proliferation of leukemia and lymphoma cells by MSCs *in vitro* is dependent on cell-cell contact and that MSCs inhibit the release of IL-10 from lymphoma cells. Our findings also indicate that when administered intravenously in a model of allogeneic BMT, MSCs can reduce GVHD without increasing tumor growth or tumor incidence. However, the mechanisms through which MSCs interact with the malignant cells remain unknown, and thus further studies on MSCs are warranted. Although the safety and clinical efficacy of MSC infusion are under evaluation in humans for the support of BMT (26–28), the benefit of using MSCs in the clinical setting still needs to be explored in prospective, controlled studies.

## Figures and Tables

**Table I:** Changes in the cell cycle in leukemia and lymphoma cells detected by flow cytometry.

Groups	Cell cycle phase		
	G_0_/G_1_ (%)	S (%)	G_2_/M (%)
A20 cells alone	31.08±1.21	62.93±1.20	5.99±0.27
MSCs:A20 = 1:1	35.86±1.26^b^	45.77±1.56^b^	18.36±2.74
FΒL3 cells alone	27.00±3.68	63.38±14.47	9.62±7.84
MSCs:FBL3 = 1:1	32.69±1.26^a^	38.40±7.25^a^	28.91±4.31
P388 cells alone	33.14±0.43	56.27±0.55	10.59±0.11
MSCs:P388 = 1:1	39.65±1.85^b^	51.03±2.10^b^	9.32±0.33

n=3.

a P<0.05,

b P<0.01 compared with the corresponding leukemia/lymphoma cells cultured alone. MSCs, mesenchymal stem cells.

**Table II:** Tumor incidence in the mice injected with A20 cells alone and MSCs plus A20 cells on day 28 following BMT.

Groups	Tumor	No tumor	Total	Incidence (%)
A20	16	1	17	94.1
MSC-A20	10	7	17	58.5^a^
Total	26	8	34	76.5

Fisher’s exact test.

a P=0.039. BMT, bone marrow transplantation; MSC, mesenchymal stem cell.

**Table III:** Distribution of T cell subsets in the peripheral blood on days 7 and 14 following BMT.

Cell culture	Time since BMT (days)	CD3^+^CD4^+^ (%)	CD3^+^CD8^+^ (%)	CD4^+^CD25^+^ (%)
A20	7	78.41±3.98	4.90±1.74	4.73±1.67
	14	4.61±0.81	34.66±14.51	0.12±0.11
MSC-A20	7	50.85±11.85^a^	37.40±9.03^b^	1.88±0.43^a^
	14	4.82±1.57	30.86±8.90	0.27±0.17

Data are presented as the means ± SEM.

a P<0.05 and

b P<0.01, in comparison to the A20 group (injected with only A20 cells) on day 7 after BMT. BMT, bone marrow transplantation; MSC, mesenchymal stem cell.
